# Leviathan: A fast, memory-efficient, and scalable taxonomic and pathway profiler for (pan)genome-resolved metagenomics and metatranscriptomics

**DOI:** 10.1101/2025.07.14.664802

**Published:** 2026-05-28

**Authors:** Josh L. Espinoza, Allan Phillips, Chris L. Dupont

**Affiliations:** 1 NewAtlantis Labs; 2 J. Craig Venter Institute; 3 Ocean BioMetrics

## Abstract

Functional profiling of metagenomes and metatranscriptomes is essential for understanding microbial community capabilities, yet current methods require computationally expensive translated-search alignments that scale poorly to the large genome-resolved reference databases now common in the field. We introduce *Leviathan*, an open-source software package for integrated taxonomic and functional profiling that operates at both genome and pangenome resolution. *Leviathan* combines *Sylph* for ultra fast alignment-free taxonomic profiling with *Salmon* for pseudo-alignment-based read quantification in DNA-space against genome-resolved gene catalogs, bypassing the translated-search step that dominates runtime in existing approaches. For each (pan)genome, *Leviathan* functional profiling produces dual metrics: pathway abundance from aggregated gene-level quantification and pathway coverage from graph-based assessment of enzymatic step completeness. On *CAMI-I* and *CAMI-II* datasets, *Leviathan* achieved up to 74-fold faster runtimes and 14-fold lower memory usage compared to *HUMAnN*, while improving genome-level assignment accuracy by up to 12% and pangenome-level accuracy by up to 5%. We demonstrate *Leviathan’s* applicability through two case studies: a marine plastisphere metagenomics dataset where differential coverage analysis revealed metabolic shifts between early and mature biofilm communities and a dental caries metatranscriptomics dataset where pangenome-resolved co-expression network analysis identified organism-specific transcriptional patterns diagnostic of health and disease states. *Leviathan* is available at https://github.com/jolespin/leviathan.

## Importance

Understanding what microbes can do, not just which ones are present, is central to translating microbiome research into actionable insight. However, functional profiling remains a computational bottleneck: existing tools rely on translated-search alignments that require extensive runtime and memory per sample. These computational demands are increasingly incompatible with the trajectory of modern metagenomics, where dataset sizes and reference genome catalogs continue to expand. *Leviathan* alleviates this bottleneck by operating in DNA-space through pseudo-alignment, delivering functional profiles up to 74-fold faster with 14-fold less memory while improving strain-level accuracy. Its native support for pangenome-resolved analysis enables researchers to routinely quantify metabolic potential and transcriptional activity at a scale and resolution that was previously impractical.

## Introduction:

High-throughput sequencing has revolutionized microbiology, enabling deep insights into the composition and function of complex microbial communities through metagenomics and metatranscriptomics. While taxonomic profiling reveals “who is there” and “how abundant they are relative to each other”, functional profiling uncovers “what they can do” providing crucial information for understanding ecosystem dynamics, host-microbe interactions, and biogeochemical cycles. Contextualizing genetic assets with sample metadata is essential for translating basic research into actionable insight with genome-resolved metagenomics as a powerful approach for building scalable science.

Taxonomic profiling can be implemented using *k*-mer based methods (e.g., *Sylph* (1), *Kraken2* (2), *Ganon* (3) requiring only a reference database of genomes or marker-based methods (*MetaPhlAn* (4), *mOTUs* (5) requiring both reference genomes and taxa-specific marker sets. While the methodologies for taxonomic profiling are well established and scalable, the development of functional profiling methodologies has lagged. Accurate and comprehensive functional profiling from meta-omics datasets requires: 1) establishing a reference catalog of (pan)genomes (ideally from *de novo* genomes recovered from said dataset) and genome-specific pathway markers; 2) profiling this reference database using FASTQ reads; 3) aggregating counts relative to each pathway within each (pan)genome; and 4) estimating the “completeness” of essential steps within each pathway for each (pan)genome (i.e., pathway coverage). These (pan)genome-resolved functional abundance and coverage artifacts are the main outputs used for downstream analysis in ecosystem-level modeling.

Existing tools such as *HUMAnN* (6) have been instrumental in establishing a reproducible framework for functional profiling in metagenomics and metatranscriptomics. Recently, *Meteor2* (7) introduced a catalog-based approach that integrates taxonomic, functional, and strain-level profiling using ecosystem-specific microbial gene catalogs, improving upon existing methods for communities well-represented by pre-compiled catalogs. However, as sequencing costs decline faster than Moore’s Law predictions, the volume of meta-omic sequencing data has grown substantially and current profiling methodologies, whether catalog-based or alignment-dependent, are not designed for the new paradigm of genome-resolved metagenomics with massive custom genome collections (*AllTheBacteria* (~2.4M genomes, https://allthebacteria.org/), *GlobDB r226* (n=306,260 dereplicated genomes) (8), *GTDB r232* (n=199,923 dereplicated genomes) (9), *OceanDNA* (n=52,325 genomes) (10), *Soil MAGs* (n=40,039 genomes) (11)). Furthermore, with a shift towards sample-specific genome-resolved metagenomics (12–14) and its dependence upon Average Nucleotide Identity (ANI) based pangenome clustering methods (12, 15, 16), the need for scalable profiling methods that can natively handle both genome- and pangenome-level resolution are essential for leveraging the power of rapid profiling techniques. With multiple strains per organism, pangenome-based approaches consider the entire gene repertoire of closely related genomes (e.g., strains clustered at a specific ANI and alignment fraction) and is essential for capturing the full functional landscape to understand functional redundancy or specialization within microbial populations (17). Neither *HUMAnN* nor *Meteor2* is designed for user-provided genome-resolved references with custom pangenome definitions, leaving this as an unmet need for researchers performing *de novo* genome-resolved metagenomics.

To address these challenges, we developed *Leviathan,* a rapid, modular, and scalable software package for integrated taxonomic and functional profiling of metagenomes and metatranscriptomes designed specifically for large *de novo* (pan)genome-resolved databases. *Leviathan* streamlines the workflows for 1) building taxonomic and functional profiling databases; 2) profiling taxonomic/sequence abundance; 3) profiling pathway abundance/coverage; and 4) merging sample-specific outputs lazily into *Xarray NetCDF* and *Apache Parquet* artifacts that can be seamlessly sliced into tabular dataframes. This work introduces the architecture and capabilities of *Leviathan* by demonstrating the significant performance improvements in accuracy, speed and memory usage when benchmarked against *HUMAnN* (6) using the standardized *CAMI I* (*Critical Assessment of Metagenome Interpretation*) low, medium, and high complexity datasets (18) and the marine CAMI II dataset (19). Finally, we demonstrate real-world case studies to stress-test *Leviathan* by analyzing a marine plastisphere metagenomics dataset and dental caries oral microbiome multi-omics dataset using metagenome-assembled genomes (MAG) with their source sequencing reads. These synthetic and real-world case studies demonstrate common paradigms in metagenomics while showcasing *Leviathan*’*s* scalability and native support for streamlined (pan)genome-level functional profiling representing a key advancement for comprehensive modern meta-omics research. *Leviathan* is an open-source software package available at https://github.com/jolespin/leviathan.

## Methods

### Benchmarking *Leviathan* and *HUMAnN*

All benchmarking and analysis was performed using a virtual machine with the following specifications: *Linux Ubuntu* 22.04 64-bit (x86_64), 30 *Intel Xeon Platinum 8358* CPU, and 222 GB memory. Benchmarking and analysis was performed using 16 threads running 2 jobs simultaneously for *Leviathan* and *HUMAnN*. The following versions were used for all benchmarking: *Sylph v0.8.1*, *Salmon v1.10.3*, *HUMAnN v3.9, Leviathan v2025.7.3, BBMap v39.28* (20), *VEBA v2.5.1,* and *PyKOfamSearch v2025.9.5.* Genome- and pangenome-level accuracies were determined by comparing the *mapped.sam* from *Salmon* for *Leviathan* and *humann_diamond_aligned.tsv* from *Diamond* for *HUMAnN*. In the case of multiple alignments in SAM files, performance was assessed using the primary alignment for determining the predicted source genome and pangenome. For specific details on commands used, please refer to Supplemental Text S1.

### Building the *Leviathan* reference index

The construction of a comprehensive reference index is a prerequisite for analysis with the *Leviathan* framework and is performed using the *leviathan-index.py* command-line executable module. This module requires a set of coding DNA sequences (CDS) in a FASTA file (*--fasta*), and a corresponding tab-separated feature mapping file (*--feature_mapping*) that links each gene ID to its functional annotations (as a Python-formatted set or list string) and its source genome ID.

The module first validates data integrity by ensuring a perfect one-to-one correspondence between gene identifiers in the FASTA and feature mapping files before compiling the metadata into efficient, serialized Python dictionaries. For gene abundance/expression quantification, the module then builds a CDS-level index using *Salmon* (21), employing the *--keepDuplicates* flag to retain all provided sequences. Concurrently, if a mapping file of genome IDs to their assembly file paths is provided (*--genomes*), the module constructs a taxonomic profiling index by generating a single sketch of all genome assemblies using *Sylph* (1), with default parameters of a 31-bp k-mer, a minimum spacing of 30, and a subsampling rate of 200. If pangenome IDs are also provided, then the pangenome information is incorporated into the index and is used for downstream profiling. Pathway-level context is integrated by either using a pre-formatted custom database or by automatically downloading and processing the *KEGG* database if the provided functional features are identified as *KEGG* orthologs (22). The module ensures relevance by verifying that features in the pathway database overlap with those in the user’s dataset. The final output is a structured directory containing the *Salmon* index, the *Sylph* sketch, the compiled databases, configuration and log files, and a summary of MD5 hashes for all components. An existing *Leviathan* reference index can be updated *post hoc* to include genome sketches by re-running the module with the *--update_with_genomes* flag. A typical command to build a new index is: *leviathan-index.py* *--fasta pathway_markers.fasta[.gz]* *--feature_mapping gene_to_ko.tsv[.gz]* *--genomes genome_paths.tsv[.gz]* *--index_directory leviathan_index/* *--n_jobs 8*

### Taxonomic and sequence abundance with *Leviathan*

The taxonomic composition of a metagenomic/metatranscriptomic sample is determined using the *leviathan-profile-taxonomy.py* command-line executable module, which leverages the alignment-free *k*-mer-based profiler *Sylph* (1). The workflow begins by generating a *k*-mer sketch from the input paired-end sequencing reads (FASTQ format). This is handled internally by executing the *sylph sketch* command with default parameters of a 31-bp k-mer size (*-k 31*), a minimum spacing of 30 between selected *k*-mers, and a subsampling rate of 200 (*-c 200*); alternatively, a pre-computed reads sketch can be provided directly. The resulting reads sketch is then compared against the reference genome database (*genomes.syldb*) previously built and stored within the *Leviathan* reference index. The core profiling is performed by the *sylph profile* command, which estimates the relative abundance of each reference genome in the sample. To ensure high-confidence assignments, we used a minimum ANI threshold of 95% (*--minimum-ani 95*) requiring a minimum of 50 shared k-mers to consider a genome present (*--min-number-kmers 50*). The module also invokes the *--estimate-unknown* flag to account for the fraction of the community not represented in the reference database which is reported under the “Sequence_abundance” variable of the resulting artifacts.

Following profiling, *Leviathan* parses the *Sylph* output, converting genome file paths back to the user-defined genome identifiers from the index. It generates two primary metrics: “Taxonomic_abundance”’, representing the relative proportion of each genome in the community, and “Sequence_abundance”, the fraction of total reads derived from each genome. If genome clusters (i.e., pangenomes) were defined during index creation, abundances are automatically aggregated to this higher level.

The final abundance tables are saved for each sample in either compressed TSV or *Apache Parquet* format at both the genome and pangenome levels. A typical command for taxonomic profiling is as follows: leviathan-profile-taxonomy.py −1 forward.fastq.gz −2 reverse.fastq.gz -n Sample_1 -d path/to/leviathan_index/ -o output_directory/

### (Pan)genome-resolved graph-based pathway completion and abundance with *Leviathan*

The functional potential and transcriptional activity of microbial communities is profiled using the *leviathan-profile-pathway.py* executable module, which integrates abundance/expression quantification with the genomic and pathway context stored in the *Leviathan* reference index. The workflow initiates by quantifying gene abundance/expression from paired-end sequencing reads. This is performed using *Salmon* (21) in its --meta mode, which is specifically optimized for accurately assigning reads in complex metagenomic samples with strain-level heterogeneity similar to metazoan single-cell isoforms. To maintain high-confidence mappings, a stringent alignment score threshold is used by default (*--minScoreFraction 0.87*), ensuring that only reads with high-quality alignments contribute to abundance estimates. The output of this step is a per-gene quantification table providing both raw read counts and Transcripts Per Million (TPM).

Following quantification, these gene-level abundances are contextualized by mapping each gene to its source genome and its annotated functional features (e.g., *KEGG* orthologs) using the metadata from the *Leviathan* reference index. The abundance/expression value of a gene annotated with multiple features is, by default, distributed equally among them, providing a scaled abundance for each feature but this can be turned off using the *--no_split_feature_abundances* option. A core feature of this module is its dual analysis of both functional potential or transcriptional activity for each pathway, resolved at the (pan)genome level.

*Leviathan* calculates pathway completion through a graph-based approach via *KEGG Pathway Profiler* where each metabolic pathway is treated as a network of functional nodes (e.g., *KEGG* orthologs). For each (pan)genome, its complete set of detected functional features are mapped onto these pathway graphs and a coverage score is computed representing the fraction of a pathway’s enzymatic steps encoded within that specific genomic context. *Leviathan* then measures the functional potential or transcriptional activity by aggregating the counts of all features constituting a given pathway within the same genomic unit. This yields a total pathway abundance value (in TPM or read counts) that reflects the collective abundance/expression of that pathway by a specific (pan)genome.

This entire analysis is performed first at the individual genome level. If the *Leviathan* reference index was constructed with genome cluster definitions (i.e., pangenomes), the module performs a subsequent aggregation, summing abundances and recalculating prevalence metrics for each cluster.

The final outputs are a series of structured tables (in Parquet or compressed TSV format) detailing gene, feature, and pathway metrics, including the dual measures of pathway abundance (activity) and coverage (potential), resolved at both the genome and, if applicable, the pangenome level. A typical command for the pathway profiling is as follows: leviathan-profile-pathway.py −1 forward.fq.gz −2 reverse.fq.gz -n Sample_1 -d path/to/leviathan_index/ -o output_directory/

### Merging sample-specific profiling into unified *Xarray NetCDF*

To facilitate study-level comparative analyses and downstream statistical modeling, *Leviathan* provides a dedicated *leviathan-merge.py* executable module that consolidates the per-sample outputs from the profiling workflows into unified, analysis-ready data structures. The input for this modules is the parent directories containing the individual sample outputs from both the taxonomic and pathway profiling modules. It systematically discovers all per-sample result files (e.g., .parquet tables) and parses them to build comprehensive, multi-sample *N*-dimensional data structures that can be accessed via lazy loading without loading the entire file into memory.

The core of this process relies on the *Xarray* library (23), which is used to construct labeled, multi-dimensional arrays that preserve the relationships between samples, genomic units (genomes or pangenomes), and features (e.g., pathways, *KEGG* orthologs). This approach intelligently groups related metrics into single, coherent data objects. For instance, when merging pathway profiling results, the module generates a single *pathways.genomes.nc* file. This artifact contains an *xarray.Dataset* where distinct data variables for *number_of_reads*, *tpm*, and *coverage* share common dimensions for *samples*, *genomes*, and *pathways*. This structure is vastly more efficient for storage and analysis than managing separate flat files for each metric which is required for the scalability and performance needed for modern datasets.

A similar aggregation is performed for all other data types, including taxonomic abundances, feature abundances, and feature prevalence, at both the genome and pangenome levels. The final outputs are persisted as compressed *NetCDF* (.nc) files, a widely-used and highly scalable format. This data model simplifies complex data wrangling, prevents data misalignment, and provides a memory-efficient foundation for streamlined access to the entire project’s results, enabling robust, integrated multi-omic analyses.

### Pathway marker detection and completeness estimation

The core innovation of *Leviathan* exists in its functional profiling engine, which synergistically combines: (i) *PyKOfamSearch* (https://github.com/jolespin/pykofamsearch) or *PyHMMSearch* (https://github.com/jolespin/pyhmmsearch), optimized implementations of *KofamScan* (22) and *HMMSEARCH* (24) utilizing *PyHMMER* (25) for rapid marker annotation; (ii) *Salmon* (Patro et al., 2017) for highly accurate and efficient pseudo-alignment of reads to gene catalogs for quantification; and (iii) the *KEGG Pathway Profiler* (https://github.com/jolespin/kegg_pathway_profiler) for robust, reaction graph-based assessment of KEGG pathway coverage and abundance. *KEGG Pathway Profiler* is an open-source reimplementation of *MGNify’s kegg-pathways-completeness-tool* (https://github.com/EBI-Metagenomics/kegg-pathways-completeness-tool) optimized for high-memory systems but could not have been developed without the fundamental work pioneered by EBI’s developers. *PyKOfamSearch*, *PyHMMSearch*, and *KEGG Pathway Profiler* have been developed to optimize annotation and are available within the *VEBA* software ecosystem (12).

### Marine Plastisphere Microbiome differential abundance and coverage

Prokaryotic and eukaryotic genomes, pangenome-assignments, and protein-coding sequences from the *Marine Plastisphere Microbiome* case study were acquired from the official repository (FigShare: 10.6084/m9.figshare.20263974) and re-annotated with updated *KEGG* orthology via *PyKOfamSearch.* Genomes, pangenomes, and CDS sequences from annotated proteins were processed using *Leviathan*. *Leviathan’s* preprocessing, index, and pathway profiling modules were used to preprocess the metagenomes, generate a unified reference index, and profile pathways, respectively.

Differential abundance between early and late-stage plastic colonizing biofilms was performed at the pangenome-level with *leviathan profile-taxonomy* relative abundances via *ANCOM-BC* (*scikit-bio* v0.7.2) in categorical mode using a pseudocount of 1 to handle zeros during log-scale transformations (26, 27). The *ANCOM-BC* method accounts for varying sampling fractions between samples and addresses multiple testing with the Holm step-down method using Bonferroni adjustments for FDR correction of P-values (*statsmodels v0.14.6*, (28)). Statistical significance of differentially abundant pangenomes was determined using FDR < 0.001.

Differential coverage between early and late-stage plastic colonizing biofilms was performed at the pangenome-level with *leviathan profile-pathway* coverage values via two-sided Mann Whitney-U *test* (*scipy v1.17.1*) (29). Rank-biserial correlation was calculated from the Mann Whitney-U test statistic and used as a measure of effect size. Holm step-down method using Bonferroni adjustments was used to account for multiple testing when assessing differential abundance and differential coverage. FDR multiple test correction of P-values was implemented both at the pangenome-level and the more stringent global level across all features. Statistically significance of differentially covered pangenome-level *KEGG* modules was determined using FDR < 0.05.

### Dental Caries Oral Microbiome

Prokaryotic genomes, pangenome assignments, and protein-coding sequences from the *Dental Caries Oral Microbiome* case study were acquired from the official study repository (FigShare: 10.6084/m9.figshare.18180614.v1) and re-annotated with updated *KEGG* orthology via *PyKOfamSearch*. The genetic assets were processed through *Leviathan* following the methods described in the Marine Plastisphere Microbiome case study above. However, only the results of *leviathan profile-pathway* at the pangenome level were used for downstream analysis to demonstrate the utility of paired metagenomics and metatranscriptomics using *Leviathan*.

Filtering of low-confidence features were filtered to only retain pangenome-level *KEGG* modules if they were 100% complete in at least 90% of the samples in either the caries-free or caries cohorts (*compositional v2023.8.28* (30)).

The *Differential Ensemble Co-expression Network* (DECN) was constructed using partial correlation with basis shrinkage as the co-expression metric (*n_draws* = 1000) with the caries-free cohort as the reference group and the caries cohort as the treatment group (31). Edges were pruned for both the positive (caries-enriched) and negative (caries-free enriched) connections using *BiDirectional Clustered Networks* (BDCN) which uses *Leiden* community detection only retaining edges that cluster together for 100% of the 1000 iterations using separate random seeds (32). Both DECN and BDCN were computed via the *ensemble_networkx v2025.7.8* Python package (33).

Positive and negative networks with signed edge weights were merged for visualization and network property calculations such as connectivity (summed connection weight) and betweenness centrality (shortest-path betweenness centrality for nodes) calculated via *NetworkX v3.6.1.* Nodes with disproportionately high betweenness-centrality were determined by using a robust adaptation of z-score as a threshold: *Median* + 3 *Median Absolute Deviance* (MAD) via *scipy*.

## Results

### Overview of *Leviathan*

**Figure 1 -:**
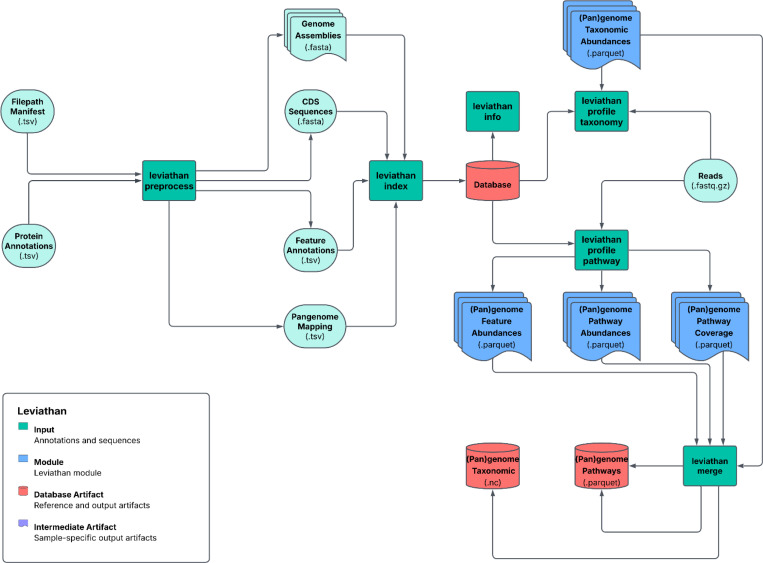
Flowchart of *Leviathan* core modules

*Leviathan* is an open-source, command-line-driven software package designed for rapid, scalable, and integrated taxonomic and functional profiling of metagenomic and metatranscriptomic data. The entire workflow is engineered for high performance and centered around a modular, three-stage architecture that streamlines analysis from FASTQ reads to multi-sample, analysis-ready data artifacts (Figure 1). *Leviathan* is run at the sample level, optimized for distributed compute environments, and the individual tabular artifacts (.parquet or .tsv) are merged into *Xarray NetCDF* files for lazy loading and efficient access to large structured database tables.

The first stage is database construction via *leviathan-index*. *Leviathan* builds a single, unified reference index containing a *Sylph* sketch for *k*-mer-based taxonomic profiling, a *Salmon* index of coding sequences for high resolution functional quantification, and pre-compiled databases that map genes to functional features (e.g., *KEGG* orthologs) and pathways. Optionally, genome clusters (i.e., pangenomes) can be provided during the database construction which will be used automatically in profiling stages. This step efficiently prepares all necessary assets for downstream analysis.

The second stage is profiling. Taxonomic profiling via *leviathan-profile-taxonomy* is essentially a convenient wrapper around *Sylph* to rapidly estimate the relative abundance of reference genomes in a sample. The core innovation of *Leviathan* exists in its functional profiling module via *leviathan-profile-pathway*, which first quantifies gene-level abundance/expression using *Salmon*’*s* meta-mode for high accuracy in complex communities. It then uniquely computes two distinct metrics for each pathway at the (pan)genome level: 1) pathway coverage, a graph-based assessment of the completeness of a pathway’s enzymatic steps, representing its genomic potential; and 2) pathway abundance, the aggregated abundance/expression of all genes within that pathway, representing its functional potential/activity.

The final stage is merging the artifacts into individual data structures via *leviathan-merge*. *Leviathan* consolidates the individual per-sample outputs into individual multi-dimensional *Xarray NetCDF* files. This creates a memory-efficient, analysis-ready data structure that contains all metrics (e.g., taxonomic abundance, pathway coverage, pathway abundance) conveniently organized by sample, feature, and genomic unit. A key design principle throughout this workflow is *Leviathan*’*s* native support for pangenome-resolved analysis, where all metrics can be seamlessly aggregated from individual genomes to user-defined pangenome clusters.

### Benchmarking Strategy

The *CAMI* datasets were used to benchmark the pathway profiling tools (18, 19). *CAMI I* and *II* datasets include 8875 genomes that cluster into 3129 pangenomes (Table 2). Of the 8875 genomes, 856 genomes were insufficient for *Pyrodigal* to identify any protein-coding genes, 3129 genomes passed quality control (*CheckM2* completeness ≥ 50% and contamination < 10%), and 7202 genomes contained high-quality *KEGG* pathway *KOfam* annotations. *Leviathan* can only incorporate genomes that have pathway annotations (i.e., *KOfam*), therefore, any genome that did not have *KOfam* annotations that exceeded the HMM threshold were excluded from the analysis. However, every genome that lacked *KOfam* annotations also failed medium stringency quality control based on *CheckM2* so they would be excluded from best-practice workflows. Within a single dataset there are multiple samples and each sample may or may not contain a separate strain of an organism. In the case of this study, each sample-specific strain is considered a genome and all genomes from a dataset (e.g., *CAMI_high_toy*) are used to build pangenomes. These genome and pangenome collections are used for building taxonomic and functional profiles. Co-assembly or pooled samples from the *CAMI* dataset were excluded to provide highly informative benchmarks with strain-level resolution.

### Native support for genome and pangenome-level profiling of microbial communities

Next-generation sequencing instruments produce data that is inherently compositional as they estimate the relative abundance of discrete biological components (e.g., transcripts, marker genes) within a community by sampling from a pool of nucleic acid fragments (30). Biological features are naturally hierarchical (e.g., genes → chromosomes/plasmids → genomes → pangenomes) and individual components can be amalgamated up the hierarchy while retaining compositionality (34). In the case of *Leviathan*, the relative abundance and counts tables produced by *Sylph* and *Salmon* in the backend, respectively, are compositional and, thus, individual component abundances can be aggregated with respect to higher order biological structures such as pangenomes and pathways. In the context of this study, a strain will be referred to as a genome and this genome is recovered from an individual sample. Each output artifact contains a genome and pangenome version allowing users to easily select which level would be appropriate for their analysis. We refer to the pathway-level output features as Taxa-Resolved Functional Modules (TRFM) where each TRFM represents the coverage and abundance of a specific metabolic pathway within a single genome or strain-dereplicated pangenome.

One caveat for estimating the abundance of a feature or pathway is that a single gene can be assigned to multiple features (e.g., *KEGG* orthologs or enzymes) and a feature can be represented in multiple pathways. To address this, *Leviathan* implements a fractional abundance for these scenarios which can be toggled off. An example of this would be *Salmon* mapping a read to *gene_a* which is annotated by 3 features {*KOfam_1, KOfam_2, KOfam_3*}. When aggregating the gene-level counts with respect to each feature, the read count would be equally distributed across the features where each feature would receive ⅓ of a count for each read mapped to *gene_a* (or ⅓ of a TPM value if using TPM as a metric). This approach ensures that the sum of feature counts equals the sum of gene counts which is necessary for interpreting relative abundances for compositional data analysis.

While *HUMAnN* technically supports pangenome analysis, the methodology for building custom databases is not user-friendly and requires users to identify marker genes that are specific to each pangenome to build *ChocoPhlAn* and *MetaPhlAn* databases. *HUMAnN*’*s* custom pangenome database construction requires taxa-specific marker identification that is not part of its standard workflow but our performance benchmarks are computed at the genome-level and accuracy calculations are performed *post hoc* so are not affected by the lack of *HUMAnN*-compatible pangenome databases.

### *Leviathan* integrates *Sylph* for rapid (pan)genome-resolved taxonomic profiling

*Leviathan* provides users with *leviathan-profile-taxonomy,* a convenient wrapper around *Sylph* which is an ultrafast and precise taxonomic profiler (1). *Sylph* is a species-level metagenome profiler that estimates genome-to-metagenome containment ANI through zero-inflated Poisson *k*-mer statistics and has superior performance relative to other approaches with support for genomes at low coverage levels (See Shaw and Yu, 2024 for benchmarking details). After running *Sylph*, *Leviathan* sums the taxonomic and sequence abundance values with respect to pangenomes and outputs relative abundance tables for genomes and pangenomes. Once the taxonomic profiling has been completed for each sample, the abundance tables are merged to build *Xarray NetCDF* artifacts where users can access data using lazy loading without storing everything into memory.

### *Leviathan* enables rapid, low-resource functional profiling

The main innovation of *Leviathan* is in the functional and pathway profiling. To quantitatively assess the performance of this approach, a direct benchmark was conducted comparing the leviathan-profile-pathway module against the widely-used *HUMAnN* method. The evaluation was performed on datasets from the *CAMI I* challenge, including samples of low, medium, and high complexity to measure performance across a range of microbial community structures. Both *Leviathan* and *HUMAnN* were executed with 16 CPU threads to ensure a fair comparison, and performance was measured by recording total wall-clock time for speed and maximum resident set size for memory usage (Table 3).

The results revealed that *Leviathan* offers a substantial improvement in computational efficiency. Across all tested datasets, *Leviathan* was up to 74-fold faster (7.3x – 74.2x) than *HUMAnN*. For example, on the high-complexity *CAMI* samples, *Leviathan* completed its functional and pathway analysis in approximately 14 minutes, whereas *HUMAnN* required between 13–18 hours for the same task. This performance advantage was coupled with a significant reduction in memory consumption. *Leviathan*’*s* peak memory usage was consistently around 14-fold lower (6.5x – 13.9x), consuming approximately 2.2 GB of RAM on the high-complexity samples compared to over 32 GB utilized by *HUMAnN*. These performance gains are attributed to *Leviathan*’*s* architecture, which leverages a pre-compiled *Salmon* index for highly efficient, alignment-free quantification and employs optimized, in-memory aggregation strategies to rapidly compute genome-resolved pathway metrics without the overhead of intermediate file parsing and alignment-based methods.

The major performance gains attributed to the pseudo-alignment approach *Leviathan* takes relative to the translated blast search that *HUMAnN* uses in the backend. More specifically, *Leviathan* employs *Salmon* to map paired-end reads directly to the CDS sequences in DNA-space while *HUMAnN* uses *Diamond* to translate reads from DNA-space into protein-space and aligns those predicted proteins to a marker protein database. Further, since *HUMAnN* leverages translated blast searches it requires users to join reads together (e.g., bbmerge.sh (20)) prior to running the pipeline as it cannot operate on paired-end reads directly. Bypassing the translated search and leveraging state-of-the-art pseudo-alignment algorithms gives *Leviathan* greater performance and accuracy.

To leverage *HUMAnN* for custom databases, users are required to align proteins to *UniRef* (35) and these proteins IDs are cross-referenced to enzymes in *MetaCyc* (36) and *MinPath* (37). However, not all the *UniRef* proteins have representatives in *MetaCyc* and *MinPath* pathways. Considering level-4 enzymes (e.g., EC-1.1.1.1), there are 1952 enzymes shared between *HUMAnN* and *MinPath*, 236 enzymes unique to *MinPath,* and 3571 unique to *HUMAnN*. For level-3 enzymes (e.g., EC-1.1.1.-), there are 186 shared enzymes, 73 unique to *HUMAnN*, and 9 unique to MinPath. These set overlaps are based on cross-referencing *HUMAnN (metacyc_reactions_level4ec_only.uniref.bz2* and *metacyc_pathways)* and *MinPath* (*MetaCyc-mapping.txt)* backend files. These findings show that many of the proteins in the *UniRef*-based custom database are not being used and are likely underestimating the true pathway coverage.

### *Leviathan* functional profiling achieves superior accuracy with strain-level resolution

The ability to accurately profile microbial communities with high strain heterogeneity is a major challenge in complex systems. *Leviathan* addresses this challenge by achieving competitive or superior accuracy on *CAMI* datasets while also maintaining optimal runtime performance. Using the same *CAMI I* toy (low, medium, and high complexity) and *CAMI II* Marine datasets, we compared the genome and pangenome assignments from the pathway abundance profiles generated by *Leviathan* and *HUMAnN* against the ground-truth profiles (Table 4).

The results revealed that *Leviathan* provides improvements in genome-level accuracy (i.e., strain resolution) while achieving near-perfect performance at the pangenome level. Across all tested datasets, *Leviathan* was up to 12% more accurate (1.5% – 12.1%) at the genome level and up to 4.9% (1.5% – 4.9%) at the pangenome-level compared to *HUMAnN*. *Leviathan*’*s* accuracy ranged from 92.3% accuracy (*CAMI II Marine sample_7*) and 99.9% accuracy (*CAMI_low_toy*) at the genome level and 98.2% accuracy (*CAMI II Marine sample_2*) and 99.9% accuracy (*CAMI_low_toy*). For the high-complexity CAMI samples, *Leviathan* consistently performed at over 94% accuracy (94.2% – 95.6%) at the genome-level and over 98% accuracy (98.7% – 99.7%) at the pangenome level. This shows that most misalignment discrepancies that align from strain heterogeneity are resolved when aggregating genome-level features to the pangenome level even in high complexity samples. In comparison, *HUMAnN* performed at ~90% accuracy (89% – 90.5%) at the genome level and ~96% accuracy (95.7% – 96.1%) at the pangenome level.

Again, the major accuracy gains are attributed to the pseudo-alignment approach *Leviathan* takes relative to the translated blast search that *HUMAnN* uses in the backend. More specifically, the use of *Salmon* and operating in DNA-space for pathway features rather than best-hit alignments in protein-space. For higher strain-level accuracy, users can specify more strict *--minimum_score_fraction* but *Leviathan* uses 0.87 which is the default for *Alevin* single-cell analysis and designed for differentiating cell-type heterogeneity (38).

### Case Study I: Marine Plastisphere Microbiome

The *Marine Plastisphere Microbiome* metagenomics cross-sectional dataset represents microbial communities from early and mature stage biofilms on plastics in marine surface water (13, 39). Of these samples, 17 early stage biofilms were collected from virgin plastic particles incubated in seawater for 2–7 days while 20 late stage biofilms were sourced from plastic litter harvested at the same locations. Genome-resolved metagenomic analysis yielded 219 prokaryotic and 5 eukaryotic MAGs clustering into 154 prokaryotic and 4 eukaryotic pangenomes. For more details regarding the experimental setup, see Bos et al., 2023.

**Figure 2 -:**
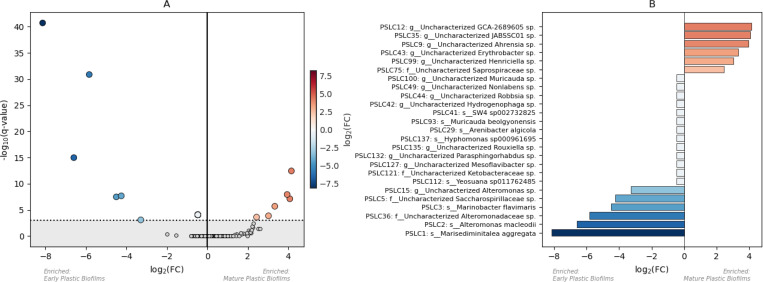
Marine plastisphere microbiome case study results from *ANCOM-BC* differential abundance test of pangenome-level taxonomic abundance. (A) Volcano plot with data points representing pangenomes (N=158) and dotted line denoting the global significance threshold (FDR < 0.001). Data points above significance threshold are enriched in the early (left) or mature (right) plastic biofilms with (B) ANCOM-BC Log2FC values shown in barchart. Taxonomic labels represent the lowest resolved rank from the consensus taxonomy of each pangenome.

To showcase the utility of *Leviathan* for metagenomics, we first investigated which microbes were enriched separately in early and mature stage plastic forming colonies. Differential abundance tests revealed 19 taxa and 6 taxa enriched in early-stage and mature-stage plastic biofilms, respectively (Figure 2, Table S1). Taxa from the family *Alteromonadaceae* were highly enriched in early-stage plastic colonies compared to mature-stage plastic colonies. Of the top 6 highly enriched taxa in the early-stage colonization, 4 were of the *Alteromonadaceae* family, *Marisediminitalea aggregata* (log_2_FC = −8.14) and *Alteromonas macleodii* (log_2_FC = −6.60) exhibiting the greatest bias-corrected log-transformed abundance, while the other enriched taxa included *Marinobacter flavimaris* (log_2_FC = −4.5) and an uncharacterized *Saccharospirillaceae* (log_2_FC = −4.3). Mature-stage plastic samples were dominated by *Alphaproteobacteria* including an uncharacterized *Hyphomicrobiaceae* (log_2_FC = 4.16), an uncharacterized *Rhodobacteraceae* (log_2_FC = 4.1), an uncharacterized *Ahrensia* (log_2_FC = 3.95), and an uncharacterized *Erythrobacter* (log_2_FC = 3.34) along with an uncharacterized *Saprospiraceae* (log_2_FC = 2.44). The differential abundance results are consistent with the plastisphere succession patterns described by Bos et al. 2023, with Alteromonadaceae-dominated copiotrophic Gammaproteobacteria in early-stage biofilms giving way to Alphaproteobacteria-dominated mature biofilm communities, including Rhodobacteraceae and Hyphomonadaceae.

To assess the variation of metabolic potential of pangenomes between early and mature-stage plastic colonizing microbes, we leveraged the functional profiling results using TRFM coverage profiles from the *leviathan profile-pathway* module at the pangenome level as they represent the metabolic capability of strain-dereplicated organisms. While the abundance of specific functional modules from metagenomics would roughly approximate the abundance of microbes, coverage can vary as microbes lose or gain genes through evolution or horizontal gene transfer. To complement the differential taxonomic abundance, we performed differential coverage analysis to investigate which TRFMs differed statistically in detected pathway step completion between early and mature plastic biofilm communities.

**Figure 3 -:**
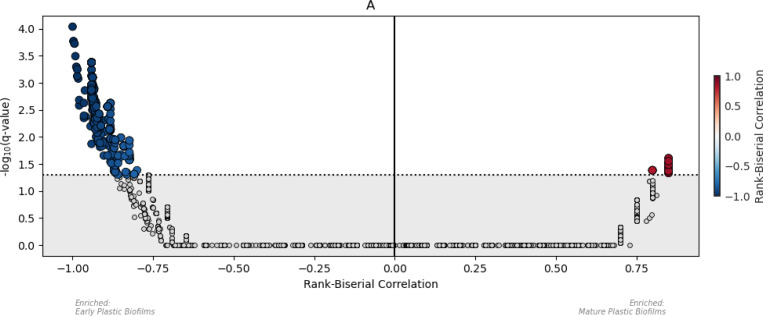

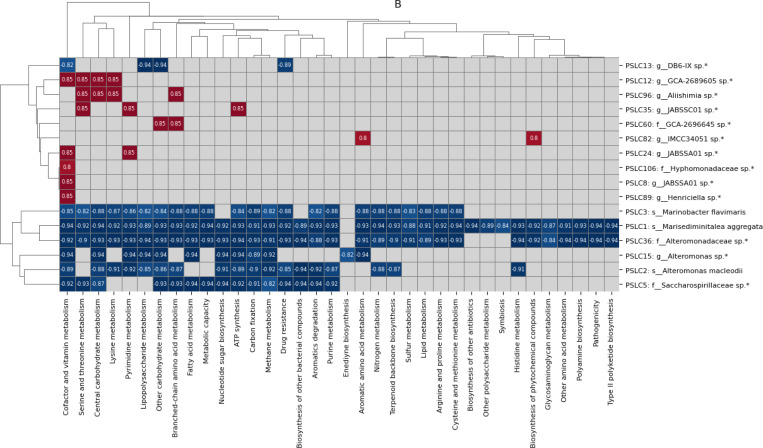
Marine plastisphere microbiome case study results from Mann Whitney-U differential pathway coverage test of pangenome-level with rank-biserial correlation. (A) Volcano plot with data points representing pangenome-specific *KEGG* modules (N=25,263) and dotted line denoting the global significance threshold (FDR < 0.05). Data points above significance threshold are enriched in the early (left) or mature (right) plastic biofilms with (B) rank-biserial correlation values averaged per pathway category shown in clustermap.

We identified 664 TRFM and 30 TRFM with statistically higher *KEGG* module completion ratios in early and mature-stage plastic colonizers, respectively (Figure 3, Table S2). Early-stage taxa, primarily dominated by *Alteromonadaceae*, exhibited broadly higher *KEGG* module completion across cofactor/vitamin metabolism (Pimeloyl-ACP biosynthesis) and central carbohydrate metabolism (gluconeogenesis and glycolysis) while mature-stage taxa exhibited higher *KEGG* module completion across amino acid metabolism (serine/threonine, lysine, and branched-chain metabolism), central carbohydrate metabolism (citrate cycle and pyruvate oxidation), and cofactor/vitamin biosynthesis (Pantothenate and Coenzyme A biosynthesis). These differential coverage patterns complement the taxonomic abundance results, demonstrating how *Leviathan*’*s* pangenome-level functional profiling can reveal metabolic plasticity that would be obscured at the individual genome level.

### Case Study II: Dental Caries Oral Microbiome

The *Dental Caries Oral Microbiome* multi-omics cross-sectional dataset represents microbial communities from supragingival plaque of Australian twin children. This study recovered 658 prokaryotic MAGs that clustered into 135 pangenomes. These genetic assets were used to build a *Leviathan* reference database and the associated 91 metatranscriptomes were leveraged to assess transcriptional patterns in caries and caries-free cohorts (31, 40).

To showcase the utility of *Leviathan* for multi-omics (metagenomics and metatranscriptomics) from an ecological perspective, we built a compositionally-valid DECN using caries-free and caries as the reference and treatment cohorts, respectively, then clustered these networks into BDCNs. Nodes in this network represent high-confidence TRFM expression (i.e., pangenome-resolved *KEGG* modules that were 100% complete in at least 90% of the cohort) and edges represent coexpression of functional modules between organisms. While co-expression of TRFM is not a replacement for robust yet resource intensive community-scale metabolic models (41), co-expression can serve as a proxy for characterizing broad transcriptional patterns that are indicative of microbial communities in the context of health and disease.

**Figure 4 -:**
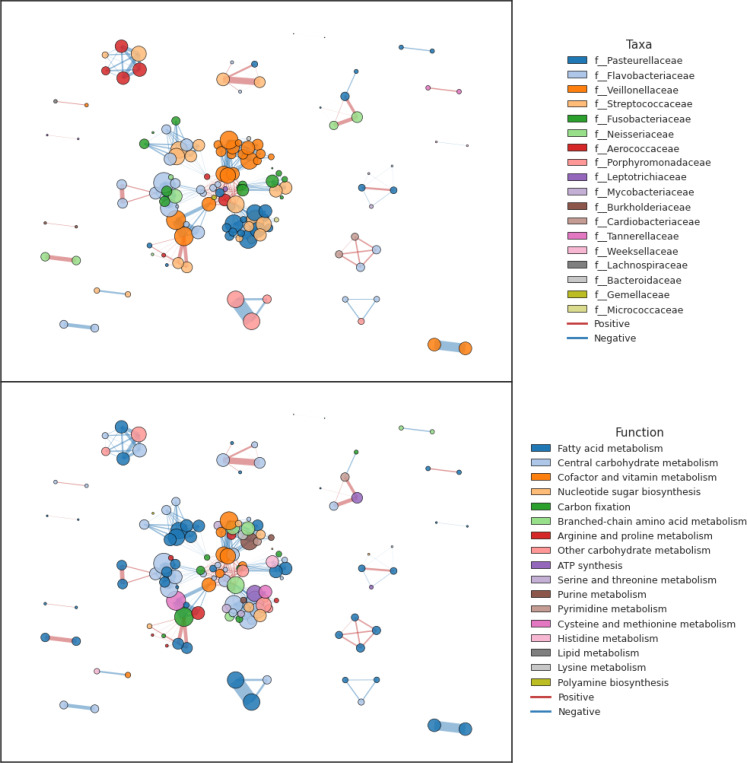
*BiDirectional Clustered Networks* TRFM connectivities enriched in caries cohort (red) and caries-free cohort (blue). TRFM nodes colored by (A) taxonomy and (B) pathway category.

The caries-enriched subnetwork clustered into 12 Leiden communities ranging from 2–12 nodes per cluster including a total of 52 nodes and 198 edges representing 24.5% and 1.71% of initial DCEN nodes and edges, respectively. The caries-free-enriched subnetwork clustered into 18 Leiden communities ranging from 2–21 nodes per cluster including a total of 108 nodes and 489 edges representing 50% and 4.2% of initial DCEN nodes and edges, respectively. The union of the caries and caries-free-enriched networks include 142 nodes with 19 intersecting nodes indicating distinct metabolism within communities and a low proportion of bridge nodes relevant to both caries and caries-free transcriptomic states (Figure 4, Tables S3,S4). The BDCN algorithm asserts that no edges overlap between the caries-enriched and caries-free-enriched clustered networks.

**Figure 5 -:**
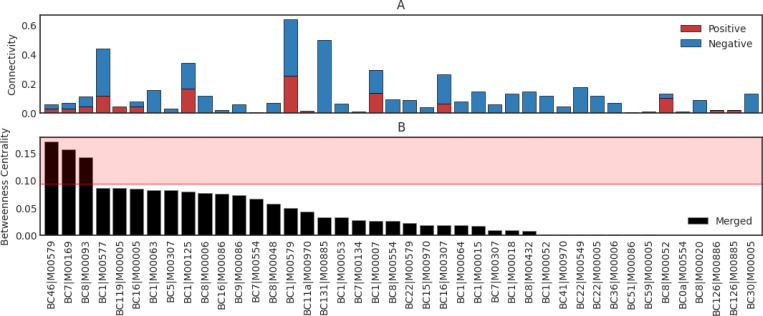
(A) Stacked barchart of *BiDirectional Clustered Networks* connectivities enriched in caries cohort (red) and caries-free cohort (blue). (B) Barchart of betweenness centrality for the union of the caries-enriched and caries-free-enriched networks with outlier bridge nodes identified using a robust outlier threshold (median + 3 MAD) of betweenness centrality.

The union BDCN reveals bridge nodes connecting modules dense with caries-enriched connections to modules dense with caries-free-enriched connections as shown in Figure 4 and the interactive Supplemental Figure 1. We used betweenness-centrality (BC) to assess which bridge nodes to interpret for demonstrating the utility of *leviathan profile-pathway* in the context of investigating caries from an ecological perspective (Table S4). Our robust adaptation of the standard z-score uses median and MAD instead of mean and standard deviation, making it resistant to outliers and therefore better suited for detecting them (Figure 5).

Analysis of betweenness-centrality revealed three distinct outliers with disproportionately high values: 1) *Granulicatella adiacens* phosphate acetyltransferase-acetate kinase conversion from acetyl-CoA to acetate (*BC46|M00579,* value=0.17*)*; 2) *Fusobacterium polymorphum* CAM pathway (*BC7|M00169,* value=0.16); and 3) *Haemophilus_D parainfluenzae* phosphatidylethanolamine biosynthesis (*BC8|M00093,* value=0.13) (Figure 5, Table S4).

The *BC7|M00169* instance brings up an important caveat regarding false positives in *KEGG Brite Hierarchy,* that is, *Fusobacterium polymorphum* is an anaerobic Gram-negative bacterium that relies on fermentation for energy in oxygen-free environments (e.g., human oral cavity) and CAM is a plant-specific carbon fixation pathway. However, there is a substantial overlap in *KEGG* orthologs between M00169 and several other *KEGG* modules including M00172 C4-dicarboxylic acid cycle (100% overlap coefficient) as well as M00171 C4-dicarboxylic acid cycle and M00173 reductive citrate cycle (50% overlap coefficient) as shown in Table S5 and documented previously (42). Thus, the source of these false positive assignments result from HMM score thresholds for *KEGG* orthology and are not an issue with *Leviathan* or the network-based analysis methods.

These three bridge TRFM appeared in both caries-enriched and caries-free-enriched subnetworks but with distinct co-expression partners. In the caries-free-enriched subnetwork, *G. adiacens M00579* co-expressed with FAS II fatty acid elongation modules in *Capnocytophaga sputigena*, *Streptococcus* spp., and *Streptococcus sanguinis*; *F. polymorphum* M00169 coexpressed with *Streptococcus* FAS II modules and its own histidine degradation and pyruvate oxidation pathways; and *H. parainfluenzae* M00093 co-expressed predominantly with its own central carbon metabolism (gluconeogenesis, pentose phosphate pathway, fumarate reductase) and *Streptococcus* glycolysis and glycogen biosynthesis. In the caries-enriched subnetwork, all three bridge nodes shifted toward inter-species coexpression with *Veillonella parvula* cofactor biosynthesis (biotin, riboflavin), *Streptococcus* branched-chain amino acid biosynthesis, and *F. polymorphum* glycogen biosynthesis, consistent with cross-feeding patterns associated with cariogenic biofilms in the ecological caries hypothesis (43).

This case study demonstrates that *Leviathan*’*s* pathway profiling, when paired with differential co-expression network analysis, can resolve organism-specific transcriptional differences between health and disease ecological states that would be undetectable with taxon-level or pathway-level profiling alone.

## Discussion

This work introduced *Leviathan*, a high-performance software package designed to address the critical computational bottlenecks in modern, genome-resolved metagenomic and metatranscriptomic functional profiling. *Leviathan* is designed for both taxonomic and functional profiling by leveraging the strengths of state-of-the-art methods such as *Sylph* (1) and *Salmon* (21) while also building on prior conceptual work pioneered by *HUMAnN* (6). *Leviathan* dramatically improves computational efficiency of functional profiling while simultaneously enhancing accuracy. The benchmarking results on the *CAMI* datasets demonstrate that Leviathan is not merely an incremental improvement but a significant leap forward, offering up to a 74-fold reduction in runtime and 14-fold decrease in memory usage compared to the widely used tool *HUMAnN*. Crucially, these gains are achieved alongside superior accuracy in resolving functional profiles at both the strain (genome) and pangenome levels where accuracy is improved up to 12% and 5%, respectively.

The substantial performance gains observed in *Leviathan* are primarily attributed to a fundamental shift in its core algorithmic strategy. Unlike *HUMAnN*, which relies on computationally expensive translated-blast searches to align reads from DNA-space into protein-space, *Leviathan* leverages the sensitivity of pseudo-alignment capabilities employed via *Salmon*. By mapping reads directly to a reference catalog of coding sequence in DNA-space, *Leviathan* bypasses the costly translation step and benefits from *Salmon*’*s* highly optimized algorithms. This architectural choice transforms functional profiling from a process that can take many hours on high-performance compute nodes into a task that can be completed in minutes on standard workstations. This democratization of high-throughput analysis lowers the barrier to entry and enables researchers to investigate larger datasets in a scalable way.

Importantly, these performance improvements do not come at the cost of accuracy as *Leviathan* demonstrates superior or competitive performance compared to *HUMAnN*. The gains in accuracy, particularly at the strain-level, can be credited to *Salmon’s meta* mode, which is specifically designed to handle the quantification of features in complex mixtures with high sequence similarity, analogous to isoform quantification in single-cell transcriptomics. This is a more nuanced approach than the “best-hit” protein alignment strategy, which can struggle to differentiate reads from closely related genomes and confirmed by the increase in accuracy of *HUMAnN* when measured at the pangenome level. These results show that while *Leviathan* achieves higher accuracy at the genome-level, any minor misassignments between closely-related strains are effectively resolved when aggregating to the pangenome-level, resulting in near-perfect accuracy. This demonstrates the robustness of the pangenome as the ideal unit for functional analysis in complex communities.

A key advance offered by *Leviathan* is its seamless, native support for pangenome-resolved analysis and flexibility for custom reference databases. While the concept of pangenome analysis is not new, its practical implementation in existing functional profiling tools is often cumbersome, requiring users to navigate complex, multi-step processes to build custom databases. *Leviathan* streamlines this entirely, integrating pangenome definitions at the indexing stage and automatically generating both genome and pangenome level outputs. This functionality makes it trivial for researchers to investigate critical biological questions related to functional redundancy, niche specialization, and the distribution of metabolic capabilities across related microbial populations.

The synergy of massive performance gains and high accuracy opens the door to previously intractable research at a global scale such as scalable biodiversity monitoring. As sequencing projects generate petabytes of data from vast environmental surveys like global ocean transects (e.g., *Tara* (44)*, GOS* (45)) or continent-spanning soil studies (11), the need for tools that can keep pace is paramount. *Leviathan*’*s* speed and low resource footprint make it possible to process thousands of metagenomes and metatranscriptomes, enabling a dynamic, near-real-time view of ecosystem function. This work contributes to the paradigm shift in cataloging “who is there” to robustly quantifying “what can they or are doing” across entire biomes at different points in time, tracking shifts in biogeochemical cycles and ecosystem responses to climate change with unprecedented resolution.

In the realm of human health, the implications are equally profound. The speed and accuracy of *Leviathan* could significantly reduce the turnaround time for clinical metagenomic analyses, moving from days to hours. Its strain-level accuracy is critical for distinguishing between pathogenic and commensal strains of the same species or for tracking the transmission of an antibiotic-resistant bacterium during a hospital outbreak. Furthermore, by enabling routine pangenome-level analysis, *Leviathan* provides clinicians and researchers with a powerful tool to survey the complete functional repertoire of a patient’s microbiota, identifying virulence factors or metabolic pathways that may be linked to disease states.

While *Leviathan* represents a significant advancement, there are areas for future development. Full functional support partially relies on *KEGG*, but the modular architecture is designed to readily incorporate other functional databases such as *MetaCyc* if pathway graphs are available which would broaden its utility. Future work will focus on expanding this database support and further optimizing data structures for even larger-scale meta-analyses.

In conclusion, *Leviathan* addresses a critical and growing need in the field of meta-omics. By delivering a solution that is orders of magnitude faster, more memory-efficient, and more accurate than current standards, it effectively removes the computational bottleneck in functional profiling. *Leviathan* makes comprehensive, pangenome-resolved functional analysis a routine, scalable, and accessible task, empowering researchers to ask more complex questions of their data and paving the way for new discoveries in both environmental science and human medicine.

## Supplementary Material

Supplement 1 **Figure S1** - Interactive network version of *BiDirectional Clustered Network* with metadata hover tool

Supplement 2 **Table S1** - Differential abundance of pangenome-level taxonomic profiling via *ANCOM-BC* of mature vs. early stage plastic biofilms.

Supplement 3 **Table S2** - Differential coverage of pangenome-level pathway profiling via Mann Whitney-U test and rank-biserial correlation of mature vs. early stage plastic biofilms.

Supplement 4 **Table S3** - *BiDirectional Clustered Network* edge weights and annotations for caries-enriched and caries-free-enriched *Differential Ensemble Co-expression Networks*

Supplement 5 **Table S4** - *BiDirectional Clustered Networks* Leiden communities, connectivities, and betweenness-centrality node-level statistics

Supplement 6 **Table S5** - Overlap coefficients between *KEGG* modules based *KEGG* ortholog sets

Supplement 7 **Text S1** - Specific details on commands used for benchmarking

## Figures and Tables

**Table 1 -:** Comparison of features between *Leviathan* and *HUMAnN*. Asterisks indicates that *HUMAnN* can support custom pangenomes but only through building custom *MetaPhlAn* and *Chocophlan* databases.

Feature	*Leviathan*	*HUMAnN*
**Alignment method**	salmon quant	diamond tblastx
**Alignment space**	DNA	Protein
**Requires MinPath**	No	Yes
**Requires taxonomic lineage**	No	Yes
**Requires translated blast**	No	Yes
**Requires UniRef for custom databases**	No	Yes
**Support custom databases**	Yes	Yes
**Supports functional profiling**	Yes	Yes
**Supports paired-end Reads**	Yes	No
Supports custom pangenomes*	Yes	Yes
**Supports taxonomic profiling**	Yes	No
**Supports stratified counts tables**	No	Yes
**Supports lazy loading data structures**	Yes	No

**Table 2 -:** Comparison of genomes from CAMI datasets

	CAMI-I			CAMI-II	CAMI
	CAMI_high_toy	CAMI_low_toy	CAMI_medium_toy	Marine	Total
***HUMAnN***	2034	30	449	5506	8019
***Leviathan***	2029	30	447	4696	7202
**Genomes With Gene Predictions**	2034	30	449	5506	8019
**Genomes Passed QC**	1280	23	255	1571	3129
**Genomes With Pathway *KOfams***	2029	30	447	4696	7202
**Total Genomes**	2036	30	450	6359	8875
**Total Pangenomes**	789	30	270	2647	3736

**Table 3 -:** Runtime performance benchmarks for pathway profiling of CAMI genomes using 16 threads

		*Leviathan*		*HUMAnN*		Fold Improvement	
		Duration (minutes)	Peak Memory (GB)	Duration (minutes)	Peak Memory (GB)	Duration	Memory
**CAMI_high_toy**	H_S001	14.61	2.34	1083.57	32.31	74.19	13.84
	H_S002	14.89	2.35	949.73	32.28	63.78	13.75
	H_S003	14.96	2.34	875.83	32.64	58.56	13.97
	H_S004	15.02	2.35	852.18	32.33	56.72	13.79
	H_S005	15.27	2.33	826.25	32.23	54.13	13.82
**CAMI_medium_toy**	M2_S001	5.78	1.62	219.25	15.95	37.96	9.83
	M2_S002	5.81	1.62	174.12	16.96	29.95	10.45
**CAMI_low_toy**	S_S001	3.29	1.27	76.90	10.00	23.40	7.87
**Marine**	sample_0	13.78	2.70	119.52	17.92	8.68	6.63
	sample_1	15.22	2.69	121.30	18.00	7.97	6.69
	sample_2	14.97	2.71	120.27	17.99	8.03	6.65
	sample_3	17.10	2.71	124.05	17.83	7.25	6.59
	sample_4	14.32	2.74	118.47	17.82	8.27	6.51
	sample_5	15.53	2.72	119.40	17.80	7.69	6.54
	sample_6	16.09	2.71	119.72	17.91	7.44	6.62
	sample_7	14.90	2.73	119.92	17.92	8.05	6.56
	sample_8	16.41	2.72	121.73	17.95	7.42	6.61
	sample_9	14.45	2.73	118.87	17.79	8.23	6.51

**Table:** 

	Accuracy	*Leviathan*		*HUMAnN*		Improvement	
Dataset	SampleID	Genome	Pangenome	Genome	Pangenome	Genome	Pangenome
**CAMI_high_toy**	H_S001	0.9492	0.9970	0.9049	0.9610	0.0442	0.0360
	H_S002	0.9551	0.9899	0.8992	0.9591	0.0558	0.0308
	H_S003	0.9556	0.9888	0.9004	0.9598	0.0553	0.0290
	H_S004	0.9496	0.9872	0.8947	0.9588	0.0548	0.0284
	H_S005	0.9420	0.9877	0.8901	0.9573	0.0519	0.0304
**CAMI_medium_toy**	M2_S001	0.9692	0.9983	0.9101	0.9620	0.0591	0.0363
	M2_S002	0.9762	0.9988	0.9177	0.9650	0.0585	0.0338
**CAMI_low_toy**	S_S001	1.0000	1.0000	0.9845	0.9845	0.0155	0.0155
**Marine**	sample_0	0.9727	0.9933	0.8783	0.9538	0.0944	0.0396
	sample_1	0.9298	0.9922	0.8793	0.9554	0.0505	0.0367
	sample_2	0.9686	0.9817	0.8768	0.9393	0.0918	0.0424
	sample_3	0.9706	0.9842	0.8596	0.9517	0.1110	0.0325
	sample_4	0.9661	0.9880	0.8454	0.9389	0.1207	0.0491
	sample_5	0.9614	0.9856	0.8740	0.9612	0.0874	0.0244
	sample_6	0.9283	0.9869	0.8684	0.9574	0.0599	0.0295
	sample_7	0.9231	0.9942	0.8719	0.9466	0.0512	0.0476
	sample_8	0.9703	0.9889	0.8764	0.9488	0.0940	0.0401
	sample_9	0.9459	0.9859	0.8657	0.9548	0.0802	0.0311

## Data Availability

*Leviathan* is an open-sourced software package available at https://github.com/jolespin/leviathan. *CAMI* datasets were downloaded from *GigaDB* (https://gigadb.org/dataset/100344). *CAMI* benchmarking and intermediate files are available on Zenodo (doi: 10.5281/zenodo.20172866). The *Marine Plastisphere Microbiome* case study metagenomes were downloaded from NCBI BioProject PRJNA777294 and source code is available at https://github.com/jolespin/leviathan-case-study-plastisphere. The *Dental Caries Oral Microbiome* case study metatranscriptomes were downloaded from NCBI BioProject PRJNA383868 source code is available at https://github.com/jolespin/leviathan-case-study-oral.
