# Supplementary material for: Leviathan: A fast, memory-efficient, and scalable taxonomic and pathway profiler for (pan)genome-resolved metagenomics and metatranscriptomics
Source: bioRxiv. 2026 May 28:2025.07.14.664802. Preprint. [Version 3] doi: 10.1101/2025.07.14.664802 (PMC13232247; doi:10.1101/2025.07.14.664802)
Supplement: Supplement 7 — Text S1 - Specific details on commands used for benchmarking [file media-7.pdf]

Text S1

Universal CAMI preprocessing

The following commands were run for low, medium, and high complexity datasets separately:

Clustering genomes into pangenomes using *skani* and *VEBA*

```
skani triangle --sparse -t 16 -o ${out_dir}/skani_output.tsv -l
${genome_filepaths} --min-af 15 -s 80 -c 125 -m 1000 --ci

cat ${output_directory}/skani_output.tsv | cut -f1-5 | tail -n +2 |
edgelist_to_clusters.py --basename -t 95 -a 50 --af_mode relaxed
--cluster_prefix "PSLC-" -o ${output_directory}/genome_clusters.tsv
--identifiers ${genome_identifiers} --export_graph
${output_directory}/networkx_graph.pkl --export_dict
${out_dir}/dict.pkl --export_representatives
${output_directory}/representatives.tsv
```

Predicting prokaryotic genes with *Pyrodigal*

```
pyrodigal -i ${fp} -a ${dir}/${id}.faa -d ${dir}/${id}.ffn -j 16 |
append_geneid_to_prodigal_gff.py > ${dir}/${id}.gff
```

Quality assessing genomes with *CheckM2*

```
checkm2 predict -i ${genome_directory} -o ${output_directory}
--threads 16 --genes -x faa --database_path ${checkm2_database}
```

Leviathan preprocessing and benchmarking *CAMI*

Identifying *KEGG Ortholog* pathway markers:

```
pykofamsearch -i ${faa} -o ${out} -p=16 -b ${db}
```

Preprocessing genomes and annotations for *Leviathan*:

```
leviathan-preprocess.py -i ${manifest} -a ${annotations} -o
${output_directory} --annotation_format pykofamsearch
```

Building the *Leviathan* index:

```
leviathan-index.py -f ${fasta} -m ${feature_mapping} -g ${genomes} -d
${index_directory} -p=16 --pathway_database ${pathway_database}
```

Taxonomic profiling with *Leviathan*:

```
leviathan-profile-taxonomy.py -1 ${r1} -2 ${r2} -n ${id} -d ${index} -p=16
-o ${output_directory}
```

Functional profiling with *Leviathan*:

```
leviathan-profile-pathway.py -1 ${r1} -2 ${r2} -n ${id} -d ${index} -p=16
-o ${output_directory} --salmon_include_mappings --alignment_format sam
```

HUMAnN preprocessing and benchmarking *CAMI*

Diamond alignment against *UniRef50*:

```
diamond blastp --db ${db} --query ${faa} --threads 4 -f 6 qseqid sseqid
stitle pident evalue bitscore qcovhsp scovhsp --max-target-seqs 1 --header
simple -o ${out}
```

UniRef50 is part of *UniRef Clusters*.

Compiling annotations for custom *HUMAnN* database:

```
compile_custom_humann_database_from_annotations.py -a ${annot} -s ${faa}
-o ${out} -t ${tax} -i ${ids} -m header
```

Building custom *HUMAnN*-compatible Diamond database:

```
diamond makedb --threads 16 --in
${output_directory}/diamond_hits.proteins.fasta --db
${output_directory}/diamond_hits.proteins${db_suffix}
```

Joining forward and reverse reads for *HUMAnN* profiling:

```
repair.sh in1=${r1} in2=${r2} out=stdout.fastq | bbmerge.sh in=stdin.fastq
int=t out=${joined_fastq} minoverlap=12 itn
```

Functional profiling with *HUMAnN*:

```
humann -i ${joined_fastq} -o ${output_directory} --threads 16  -v
--search-mode ${db_name} --memory-use minimum --input-format "fastq.gz"
--bypass-nucleotide-search --diamond ${DIAMOND_PATH} --evalue 1.0
```

```
--protein-database ${db_directory} --output-basename humann --id-mapping
${id_mapping} --o-log ${output_directory}/humann.log
```

repair.sh and bbmerge.sh are provided in the *BBMap* software suite
